# A Novel Mode of Photoprotection Mediated by a Cysteine Residue in the Chlorophyll Protein IsiA

**DOI:** 10.1128/mBio.03663-20

**Published:** 2021-02-16

**Authors:** Hui-Yuan Steven Chen, Dariusz M. Niedzwiedzki, Anindita Bandyopadhyay, Sandeep Biswas, Himadri B. Pakrasi

**Affiliations:** a Department of Energy, Environmental and Chemical Engineering, Washington University, St. Louis, Missouri, USA; b Department of Biology, Washington University, St. Louis, Missouri, USA; KUMC

**Keywords:** photosynthesis, cyanobacteria, photoprotection, *Synechocystis*, energy dissipation

## Abstract

Oxygenic photosynthetic organisms have evolved a multitude of mechanisms for protection against high-light stress. IsiA, a chlorophyll *a*-binding cyanobacterial protein, serves as an accessory antenna complex for photosystem I. Intriguingly, IsiA can also function as an independent pigment protein complex in the thylakoid membrane and facilitate the dissipation of excess energy, providing photoprotection. The molecular basis of the IsiA-mediated excitation quenching mechanism remains poorly understood. In this study, we demonstrate that IsiA uses a novel cysteine-mediated process to quench excitation energy. The single cysteine in IsiA in the cyanobacterium *Synechocystis* sp. strain PCC 6803 was converted to a valine. Ultrafast fluorescence spectroscopic analysis showed that this single change abolishes the excitation energy quenching ability of IsiA, thus providing direct evidence of the crucial role of this cysteine residue in energy dissipation from excited chlorophylls. Under stress conditions, the mutant cells exhibited enhanced light sensitivity, indicating that the cysteine-mediated quenching process is critically important for photoprotection.

## INTRODUCTION

Exposure of cyanobacteria, algae, and green plants to high light intensities often leads to damage to their photosynthetic apparatus. Cyanobacteria are oxygenic photosynthetic prokaryotes that depend on sunlight for their growth and survival. During billions of years of their evolution, these microbes have developed a number of strategies to modulate light absorption and dissipation to ensure maximal photosynthetic productivity and minimal photodamage to cells under extreme light and limiting nutrient conditions. Iron deficiency is a common nutrient stress in various cyanobacterial habitats (1–5). In cyanobacteria, iron is mostly used in photosynthetic reaction center complexes and in iron-depleted environments; their photosynthetic machinery exhibits certain adaptive changes such as decreased amounts of chlorophyll (Chl)-binding proteins and phycobilisomes (6–8). Another adaptive photoprotective strategy that cyanobacteria have evolved is the induction of iron stress-induced protein A (IsiA) (7, 9).

IsiA is a Chl *a*-binding membrane protein that was first discovered in cyanobacteria grown in iron-free media (7, 9). Later studies showed that IsiA can also be induced by oxidative stress, high salt, heat stress, and, in particular, high light (10–15). IsiA belongs to a superfamily of antenna proteins with six transmembrane helices (16) and is highly homologous to CP43, an intrinsic antenna protein of photosystem II (PSII). However, unlike CP43, IsiA is mainly found to be associated with photosystem I (PSI) and forms PSI_3_-IsiA_18_ supercomplexes (17–19). Time-resolved spectroscopic studies showed that the energy transfer from IsiA to PSI and between IsiA copies in PSI-IsiA supercomplexes is fast and efficient (20, 21). Since 1 IsiA molecule binds 17 Chl *a* molecules (19, 21, 22) and 1 PSI monomer binds 96 Chl *a* molecules (23), the outer IsiA ring can theoretically increase the absorption cross section of the PSI_3_-IsiA_18_ supercomplex by at least 81% compared to a PSI trimer alone. *In vivo*, IsiA increases the effective absorption cross section of PSI by ∼60% (24). These results showed that in the PSI_3_-IsiA_18_ supercomplex, IsiA serves as an accessory antenna for PSI.

In addition to the PSI_3_-IsiA_18_ supercomplex, IsiA-only pigment protein complexes have been observed in cyanobacterial cells after prolonged growth under iron-depleted conditions (25) as well as under high-light conditions (15). Such IsiA-only complexes have been suggested to be involved in nonphotochemical quenching processes (26). Previous studies showed that an *isiA* deletion strain is more light sensitive, suggesting that IsiA plays a significant role in providing photoprotection (12, 26, 27). Using time-resolved fluorescence (TRF) spectroscopy, it has been determined that the accumulation of IsiA-only complexes in the cells results in strong quenching of IsiA fluorescence, suggesting its photoprotective role (27, 28). However, the mechanism of IsiA-mediated excitation quenching is not fully understood. Previously, it was proposed that the carotenoids present in the IsiA protein are solely responsible for the quenching of the singlet state of Chl *a* (29, 30). However, this premise has recently been questioned, and instead, we have proposed a novel cysteine-mediated quenching mechanism for this cyanobacterial pigment protein (31). Such a mechanism was originally proposed for the Fenna-Matthews-Olson (FMO) protein complex in anoxygenic green sulfur bacteria (32).

**FIG 1:**
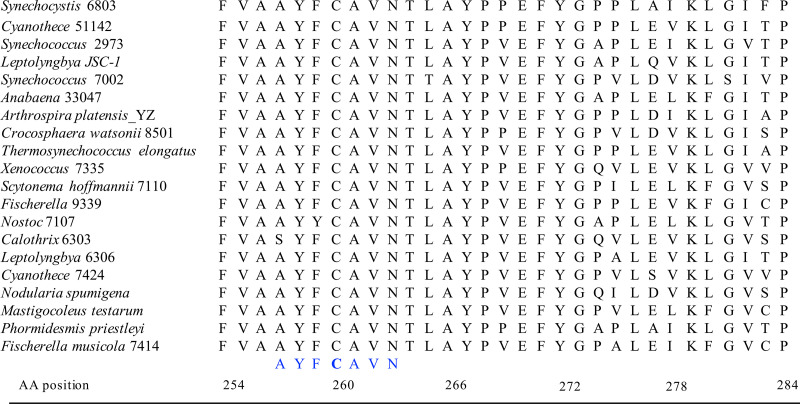
Sequence alignment of the IsiA protein showing the conserved cysteine residue. The sequence alignment shows the IsiA proteins from 24 strains representative of unicellular, filamentous, diazotrophic, and nondiazotrophic cyanobacteria. The sequences were aligned with ClustalW within MEGA 7 (67). The cysteine residue in the AYFCAVN motif is conserved across the cyanobacterial strains. AA, amino acid.

A recent single-particle cryo-electron microscopy study reported the structure of the PSI_3_-IsiA_18_ supercomplex from *Synechocystis* sp. strain PCC 6803 (here *Synechocystis*) at a 3.5-Å resolution (19). This pioneering study has provided significant insights into the protein-protein interactions in the PSI-IsiA supercomplex and identified the potential terminal emitters, the Chl *a* molecules located at the PSI-IsiA interface, in IsiA (19). Since then, two other studies have elucidated the structures of IsiA in PSI-IsiA complexes from two other cyanobacterial species at even higher resolutions (33, 34). Although a high-resolution structure of an IsiA-only complex is yet to be determined, the available structures of IsiA in the PSI-IsiA supercomplex help shed new light on the excitation energy quenching process in this protein. *Synechocystis* IsiA has a unique cysteine, C260, which is part of an “AYFCAVN” motif (35) located at the luminal side of transmembrane helix V (19). An alignment of the amino acid sequences of IsiA from several representative cyanobacterial strains shows that this motif is highly conserved in cyanobacteria (Fig. 1). Interestingly, in CP43, a protein that does not quench excitation on its own, the cysteine residue in this conserved motif is replaced by valine. According to the proposed cysteine-mediated quenching mechanism (31), a valine at that position would be unable to facilitate the quenching process. In the current study, we performed site-directed mutagenesis to construct *Synechocystis* strains in which the unique cysteine in IsiA was replaced with a valine. Remarkably, with this single-amino-acid change, mutant IsiA was unable to quench excitation energy but was still capable of serving as an efficient light-harvesting antenna for PSI. Furthermore, the C260V mutant strain was more light sensitive under stringent iron-depleted conditions but had a higher growth rate than the wild-type (WT) cells under iron-replete conditions under high light.

## RESULTS

### Construction of C260V and C260V-His *Synechocystis* strains.

The C260V mutation was introduced into the WT *Synechocystis* strain using a CRISPR/Cas12a (Cpf1) system (36). The resulting C260V strain is a markerless mutant with the cysteine-to-valine substitution as the only change. All the physiological comparisons in this study were done using this mutant and WT *Synechocystis* strains. On the other hand, for the biophysical and biochemical studies, pure IsiA and PSI-IsiA supercomplexes were needed. In our previous study, a histidine-tagged IsiA transgenic line was generated, which enabled us to purify the individual supercomplexes (31). In this study, the C260V mutation was introduced into this IsiA-His strain via double homologous recombination. The resulting strain, C260V-His, was grown under iron-depleted conditions that induce *isiA* expression. The mutant IsiA and PSI-IsiA supercomplexes were purified from the C260V-His strain by affinity chromatography followed by rate-zonal centrifugation.

### Biochemical and spectroscopic analyses of mutant PSI-IsiA and IsiA protein complexes.

**FIG 2:**
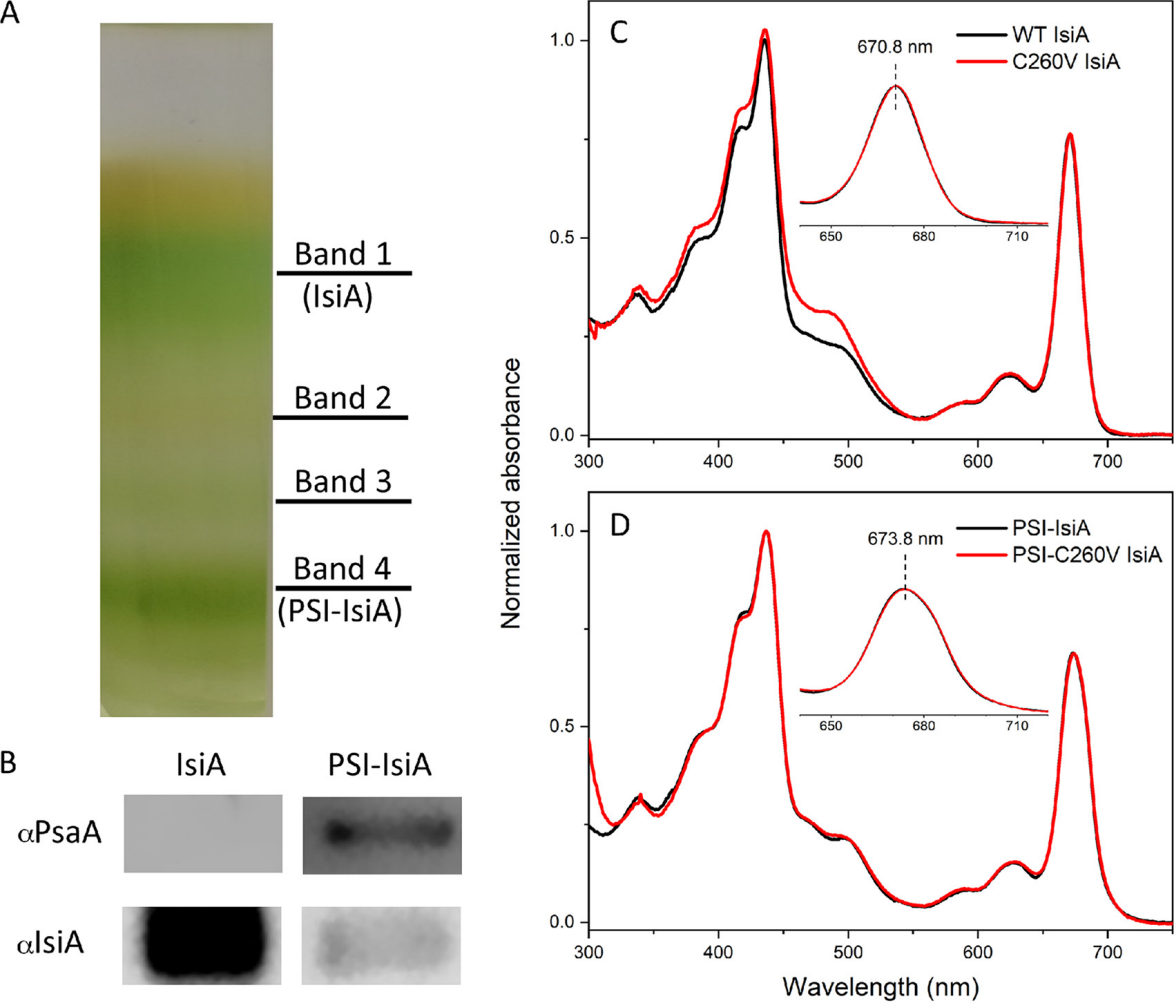
Purification of C260V IsiA and PSI-C260V IsiA pigment protein complexes from the C260V-His-tagged strain and their basic spectroscopic characterization. (A) Pigment protein bands obtained from sucrose gradient ultracentrifugation, with the IsiA and PSI-IsiA bands labeled. (B) Analysis of IsiA and PSI-IsiA sample purity by immunoblotting using antisera raised against PsaA (αPsaA) and IsiA (αIsiA), respectively. (C and D) Room-temperature absorption spectra of WT and C260V IsiA (C) and isolated WT and C260V PSI-IsiA complexes (D).

To assess how the single-amino-acid substitution C260V affects the biophysical properties of the mutant PSI-IsiA and IsiA aggregates, pure IsiA and PSI-IsiA supercomplexes were isolated. Figure 2A shows the purified protein complexes. Band 1 (top) and band 4 (bottom) were analyzed by immunoblotting. The proteins in both bands were fractionated by sodium dodecyl sulfate-polyacrylamide gel electrophoresis (SDS-PAGE) and probed using antisera raised specifically against PsaA and IsiA (Fig. 2B). The results showed that band 1 contained the IsiA-only complex without any PSI contamination, whereas band 4 contained PSI-IsiA supercomplexes. These samples are abbreviated as C260V IsiA and PSI-C260V IsiA, respectively. Sample purity was also confirmed by analysis of room-temperature absorption spectra of both preparations (Fig. 2C and D). Comparison of the spectroscopic profiles of WT IsiA and C260V IsiA shows that both complexes have essentially identical Chl *a* Q_y_ bands with an absorption maximum at 670.8 nm (Fig. 2C), indicating no PSI contamination, which is known to shift the position of the Chl *a* Q_y_ band by a few nanometers toward longer wavelengths (22, 31, 37). Such a shift was observed in the spectra of both WT and mutant PSI-IsiA complexes (673.8 nm) (Fig. 2D). In addition, the higher absorbance in the 450-nm to 500-nm region indicated an enhanced level of carotenoids in the C260V IsiA sample. This may be a physiological response to the mutation.

### Chl *a* fluorescence decay dynamics in WT and C260V IsiA complexes.

**FIG 3:**
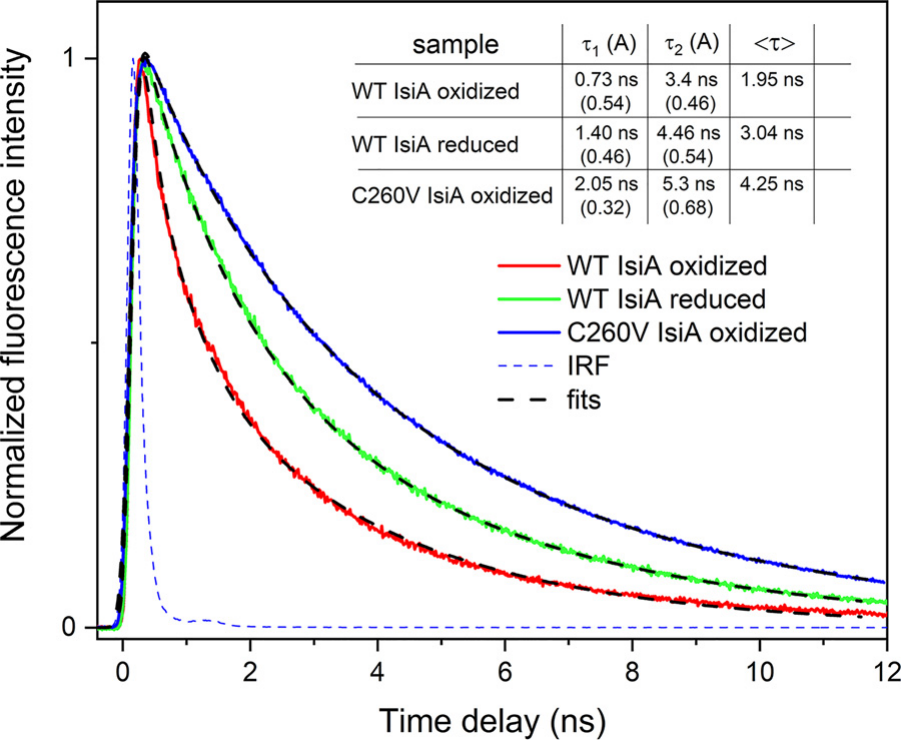
Fluorescence decay dynamics of IsiA-bound Chl *a* in WT and C260V strains under oxidative (buffer as-is) and reducing (after the addition of 10 mM sodium dithionite) conditions. Fluorescence decay was recorded at 684 nm at room temperature. IRF, instrument response function. The inset table shows fitting results with lifetimes and amplitudes of contributing kinetic components as well as the amplitude-weighted lifetime, <τ>. The signals were normalized for better comparability.

Our previous studies of Chl *a* fluorescence decay in WT IsiA demonstrated that the Chl *a* fluorescence lifetime is sensitive to the presence of the reducing agent sodium dithionite in the sample buffer (31). Therefore, we hypothesized that Chl *a* lifetime extension is caused by an inhibition of cysteine-mediated excitation quenching and reasoned that without the unique cysteine residue, this quenching mechanism in IsiA would be significantly affected or even completely absent, giving rise to longer Chl *a* fluorescence decay in the C260V IsiA mutant. This hypothesis was verified as demonstrated in Fig. 3. Our comparison of Chl *a* fluorescence decay in WT and C260V IsiA samples (Fig. 3) showed that the fluorescence lifetime of mutant IsiA is even longer than that of chemically reduced WT IsiA, indicating the absence of excitation energy quenching in the mutant strain.

**FIG 4:**
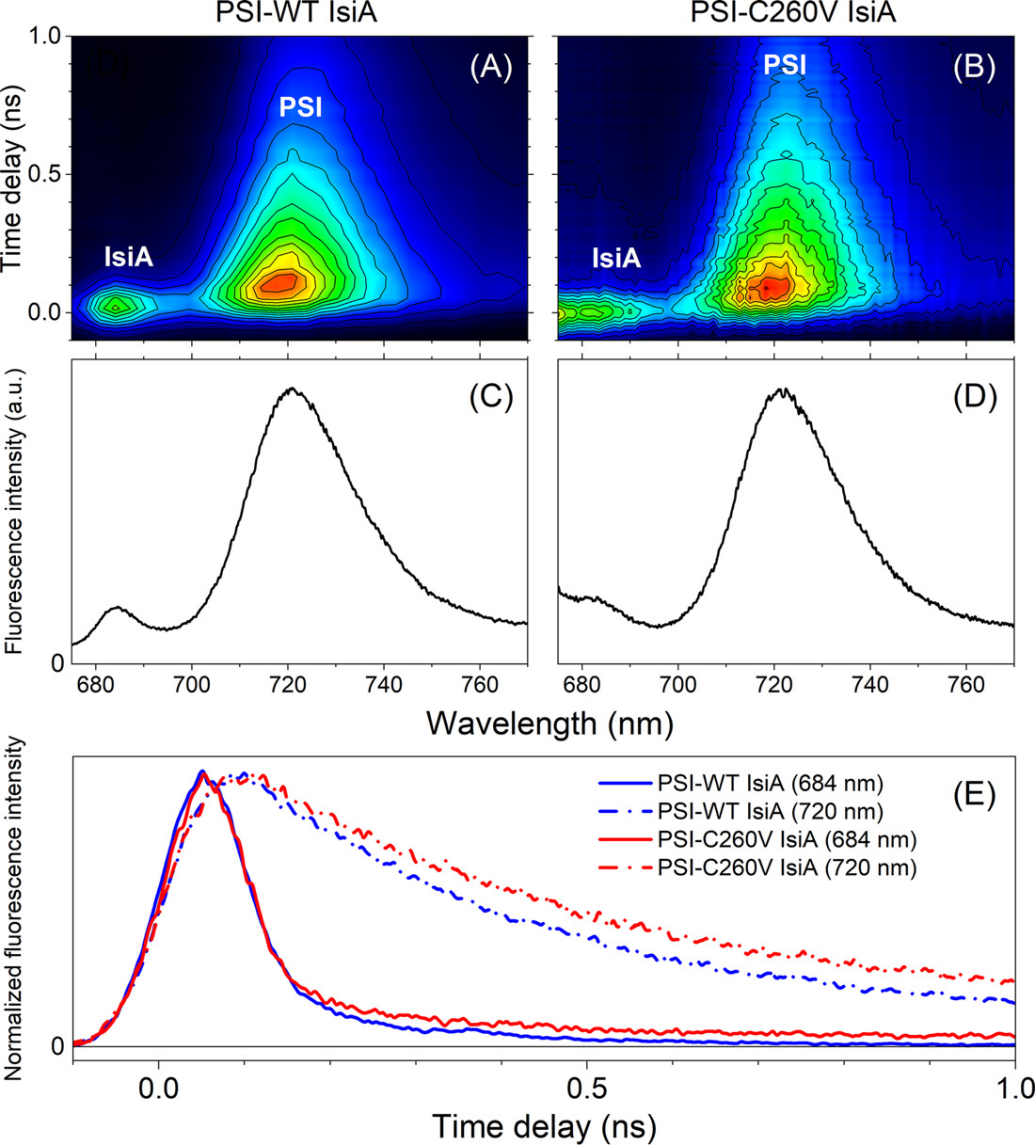
Time-resolved fluorescence from PSI-IsiA supercomplexes at 77 K. (A and B) Two-dimensional pseudocolor fluorescence decay profiles of PSI-WT and PSI-C260V IsiA supercomplexes. (C and D) Time-integrated spectra that correspond to steady-state fluorescence emissions from both supercomplexes. a.u., arbitrary units. (E) Comparison of IsiA-bound Chl *a* fluorescence decays in both samples. The kinetic traces are normalized to their maxima for better comparability. The samples were excited at 660 nm.

Next, time-resolved fluorescence spectra revealed that both WT and C260V IsiA proteins, when assembled into supercomplexes with PSI, show substantial shortening of Chl *a* fluorescence decay, demonstrating that the mutant antenna complex was capable of efficient transfer of excitation energy to PSI (Fig. 4). Figure 4A and B show two-dimensional pseudocolor fluorescence decay profiles recorded for both supercomplexes. Cryogenic temperature allows the recording of fluorescence from PSI (720-nm band), and therefore, our measurements were performed at 77 K. Figure 4C and D show time-integrated fluorescence spectra that were generated by the integration of all of the time-resolved spectra and, in principle, should be equivalent to the steady-state fluorescence spectra of the supercomplexes. Small differences visible in the spectral profiles (670 to 680 nm) and the residual long-lived signal at ∼680 nm in the mutant sample are associated with larger scattering of the excitation beam and possible residual contamination with free Chl *a.* Fluorescence decay traces of Chl *a* associated with IsiA and PSI, normalized to unity at their maxima (Fig. 4E), revealed that fluorescence decays of IsiA-bound Chl *a* were similar in both WT and C260V samples. Therefore, both WT and mutant IsiA function equally well as light-harvesting antennae and donors of excitation energy to PSI.

### Changes in the composition of pigments and key membrane proteins in the C260V mutant and WT *Synechocystis* strains.

**FIG 5:**
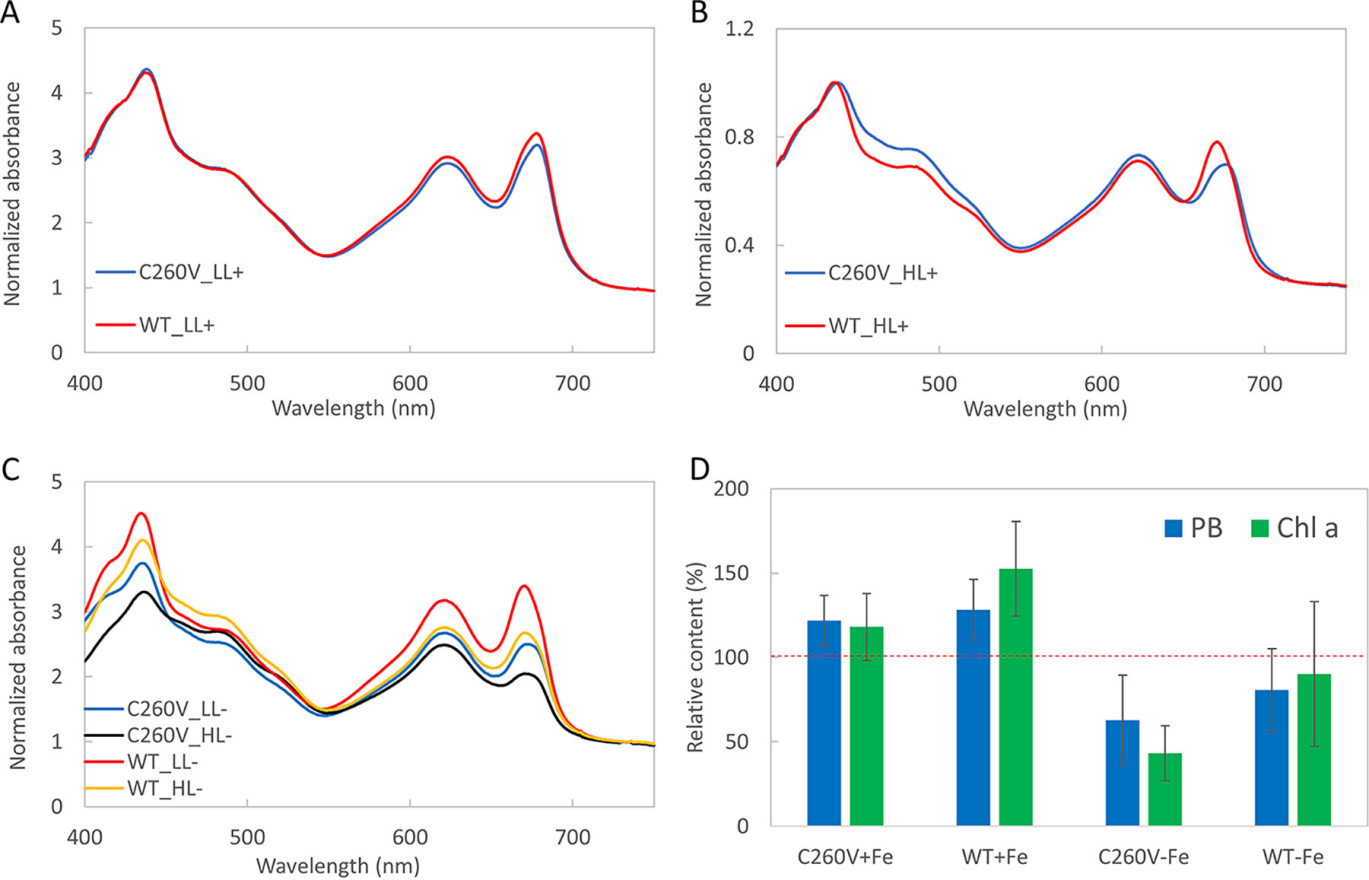
Absorption spectra and relative pigment contents of WT and C260V strains. (A to C) Cultures were grown in multicultivators under 200 μmol photons m^−2^ s^−1^ (low light [LL]) with sufficient iron (+) (A), under 800 μmol photons m^−2^ s^−1^ (high light [HL]) with sufficient iron (+) (B), and under low light and high light in the absence of iron (−) (C). (D) Relative phycobilin (PB) and Chl *a* contents per cell in WT and C260V strains under iron-replete and iron-depleted conditions. The spectra were normalized to the absorption at 730 nm. The pigment content of both strains grown under low light is represented as 100% (red dashed line), and the bars represent the phycobilin and Chl *a* contents of cultures grown under high light. Error bars represent standard deviations across triplicate biological samples.

To assess the impact of the C260V mutation on cellular physiology, we analyzed pigment and protein compositions of the mutant cells grown under different conditions. The absorption spectra of C260V and WT cultures grown under low-light and iron-replete conditions had no noticeable difference (Fig. 5A). This was expected because *isiA* is not expressed under these conditions, and therefore, the mutation is not likely to affect the physiology of the cells. In contrast, when grown under high-light and iron-replete conditions, *isiA* expression was induced in both cultures, as indicated by the blue shift of the Q_y_ absorption band (Fig. 5B) (35). The absorption spectra of cells grown under low- as well as high-light and iron-depleted conditions show that both strains have the blue shift of the Chl *a* Q_y_ absorption band from 678 nm to 671 nm. Interestingly, when the C260V mutant is subjected to high light and iron stress, the absorption band at 671 nm is significantly reduced, implying a decreased Chl *a* content (Fig. 5C). In addition, the Chl *a* Q_y_ band is markedly lower than the phycocyanin peak at 625 nm, suggesting an altered pigment composition in the mutant strain.

To further investigate the impact of high light on mutant cell physiology, we compared the pigment and protein compositions of the cells grown under high as well as low light intensities (Fig. 5D). Under high-light and iron-replete conditions, the C260V mutant showed an ∼20% increase in both phycobilin and Chl *a* contents, while the WT showed an ∼30% increase in phycobilin and an ∼50% increase in Chl *a* contents. On the other hand, under high-light and iron-depleted conditions, phycobilin and Chl *a* contents in both strains decreased pronouncedly. Most striking was the change in the Chl *a* content of the C260V mutant, an ∼55% reduction. The higher phycobilin-to-Chl *a* ratio in the C260V mutant under high-light and iron-depleted conditions (Fig. 5C) could be attributed to this severe reduction in Chl *a* content. This led us to investigate which of the Chl *a*-binding proteins were specifically lost to cause such a remarkable decrease in the Chl *a* content.

**FIG 6:**
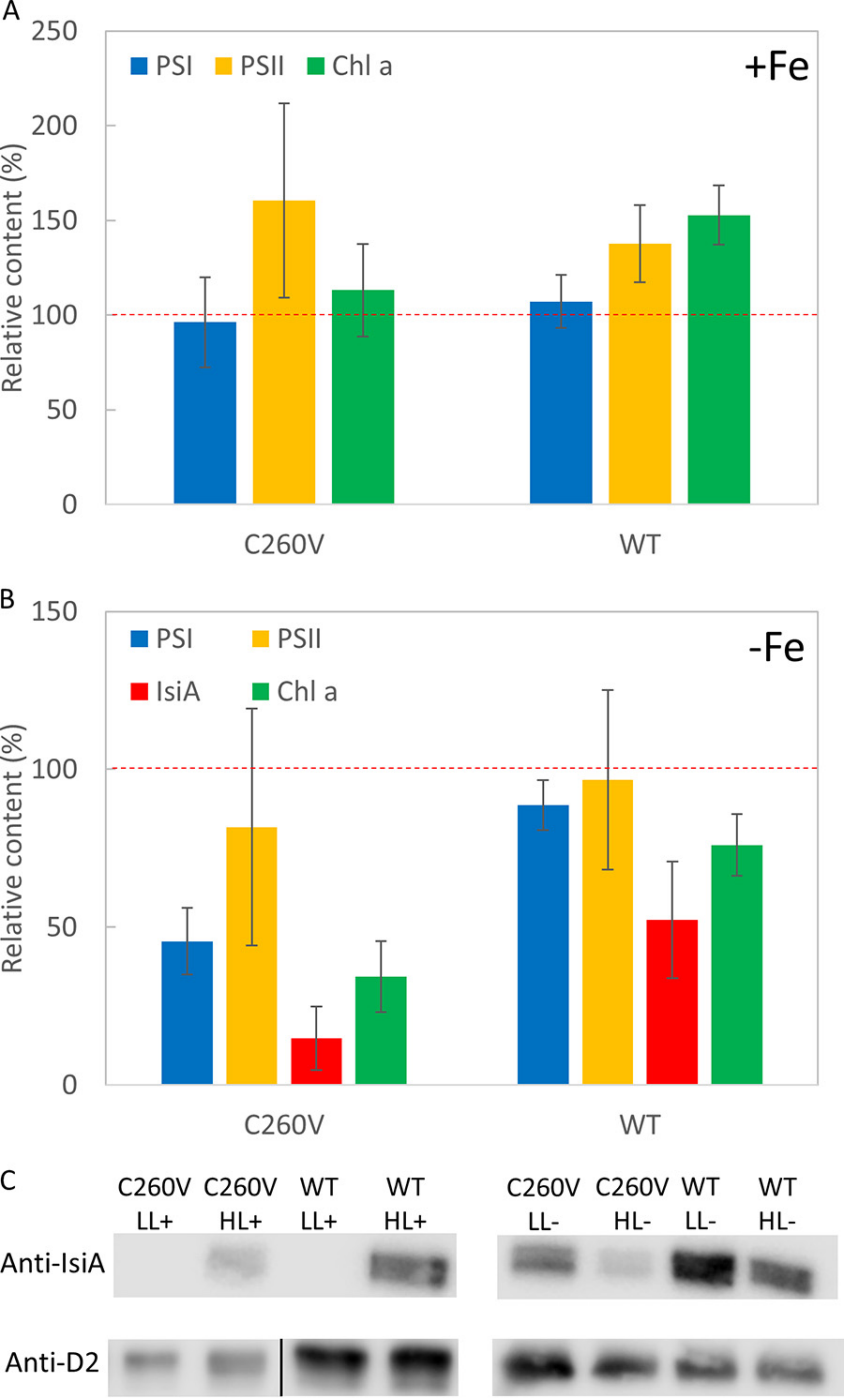
Relative abundances of Chl *a*, IsiA, and photosystems of WT and C260V mutant strains. (A and B) Chl *a*, IsiA, PSI, and PSII contents of C260V and WT cells grown in iron-replete (A) and iron-depleted (B) media. The protein and Chl *a* contents of both strains grown under low light (LL) are represented as 100% (red dashed line), and the bars represent the relative protein and Chl *a* contents of cells grown under high light (HL). (C) Immunoblot analysis of thylakoid membranes from C260V and WT cells grown under iron-replete conditions and low light (LL+), iron-replete conditions and high light (HL+), iron-depleted conditions and low light (LL−), and iron-depleted conditions and high light (HL−). Samples were probed with specific antisera against IsiA and D2. Error bars represent standard deviations across triplicate biological samples.

Under high-light and iron-replete conditions, both the C260V mutant and WT cells showed increases in PSII and Chl *a* contents (Fig. 6A). Neither strain expressed *isiA* when grown under low light in the presence of iron, and hence, the relative IsiA content under these conditions is not shown (Fig. 6A). In contrast, when grown under high light, both strains produced IsiA (Fig. 6C). However, the high-light-induced increase in the Chl *a* content was not as marked in the C260V mutant as in the WT strain, while the C260V mutant exhibited a higher PSII content. This is because of the much higher IsiA content in the WT cells. Interestingly, under iron-depleted conditions, the PSI, IsiA, and Chl *a* contents of the C260V mutant decreased significantly (Fig. 6B), whereas the WT cells showed a slight decrease in the PSI and PSII contents and a more noticeable decrease in the IsiA content, causing a moderate decrease in the total Chl *a* content (Fig. 6B). In addition, the ratio of the IsiA content to the PSI content in the C260V mutant became much lower than that in the WT strain, suggesting a significant decrease in the IsiA-only complex in the C260V mutant under high-light conditions due to the mutation. These findings indicate that the lack of the quenching ability of the mutant IsiA protein under iron-depleted and high-light conditions resulted in severe photodamage of IsiA and PSI.

### Growth of C260V mutant and WT *Synechocystis* strains under high light and iron stress.

**FIG 7:**
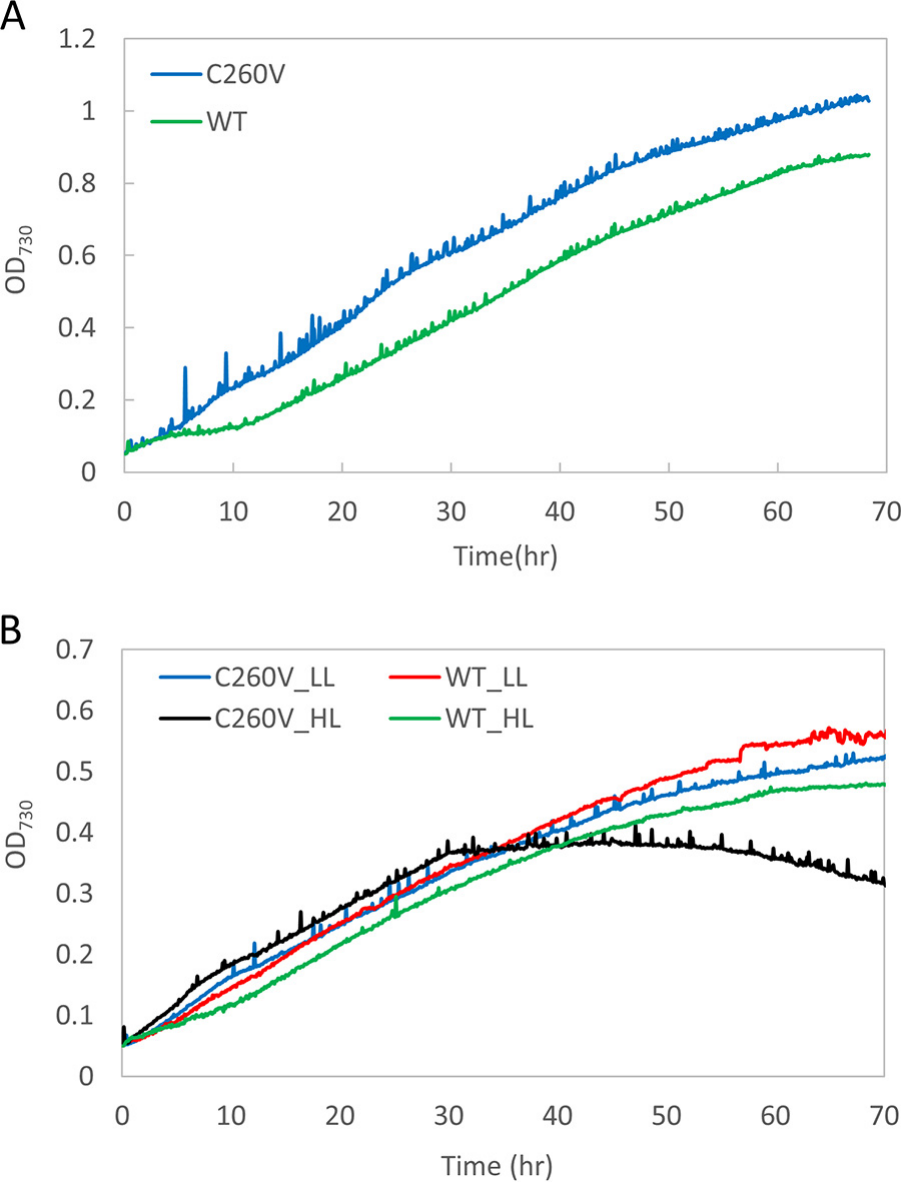
Comparison of growth patterns of WT and C260V strains. Shown are the growth curves of C260V and WT cultures under iron-replete conditions under 800 μmol photons m^−2^ s^−1^ (high light [HL]) (A) and iron-depleted conditions (with the addition of the iron chelator DFB) under low light (LL) and high light (B).

To elucidate the effect of the single-amino-acid change on cell growth, we compared the growth rates of the C260V mutant and WT *Synechocystis* strains under different conditions (Fig. 7A and B). Although the growth rates of the two strains were not significantly different from each other, differences in their growth patterns were evident. The initial lag phase was missing in the mutant strain, and consequently, it grew faster during this phase. Under high-light and iron-replete conditions, the mutant strain grew significantly faster and reached a higher optical density at 730 nm (OD_730_) in 3 days than the WT strain (Fig. 7A). This suggested that with sufficient iron in the growth medium, the lack of IsiA-mediated excitation quenching helped accelerate the growth of the mutant cells. Next, to remove trace amounts of iron remaining in the cells that were used as an inoculum, deferoxamine (DFB), an iron chelator, was added to create stringent iron-deficient conditions. Under such severe iron deficiency and high-light conditions, the mutant did not show a lag phase and exhibited an initially higher growth rate than the WT strain. However, there was a decline in growth after about 30 h, when the cells also showed a bleached phenotype (Fig. 7B). In contrast, under low light, the mutant did not exhibit a lag phase and grew as well as the WT without bleaching out. These results showed that the C260V mutant is significantly more light sensitive than the WT strain under severe iron-limited conditions and demonstrated the significant role that the Cys-mediated quenching mechanism in this protein plays in cellular photoprotection.

## DISCUSSION

Excessive high light intensities can lead to damage to the cellular machinery of cyanobacteria, algae, and plants (38). Various mechanisms have evolved in cyanobacteria to avoid such light stress. One such process, unique to these prokaryotes, is based on an orange carotenoid protein (OCP) that can directly quench the excess excitation in the phycobilisomes. The mechanism of this process has been carefully dissected during recent years (39, 40). Here, we have described another novel and potentially more universal mechanism of photoprotection based on excitation quenching mediated by a Cys residue in the Chl antenna protein IsiA.

### Energy transfer in the mutant C260V IsiA protein.

There are three proposed physiological roles of IsiA in cyanobacterial cells. First, in a PSI-IsiA complex, IsiA acts as an efficient accessory antenna for PSI (20, 21, 24, 41). Second, IsiA plays a significant role in dissipating excess light energy and thus acts as a cellular photoprotectant (12, 25–28, 42). Third, under stress conditions, IsiA acts as a Chl reservoir and becomes an immediate source of chlorophylls when such stress is relieved and new photosystem complexes are formed (43–46). In any case, the IsiA-only pigment protein complex needs to have an energy dissipation system to avoid deleterious effects of overexcitation and harmful radical formation. In this context, carotenoids in IsiA are in proximity to some Chl *a* molecules (19) and could play a role in excitation energy quenching, as previously proposed (30). However, in a recent study, we conclusively demonstrated that excitation energy transfer between Chl *a* and carotenoids in IsiA does not occur (31). Thus, there is a need for an alternate mechanism for quenching of excitation energy in the IsiA complex.

Based on chemical redox titration experiments, we previously suggested that IsiA uses a cysteine-mediated mechanism, similar to that in an FMO complex, to quench excitation energy (31, 32). It has been proposed that in FMO under oxidizing conditions, quenching of excitation energy in bacteriochlorophyll *a* (BChl *a*) is facilitated by electron transfer between the excited BChl *a* and the thiyl radical at a cysteine residue (32). The rate of photosynthesis is thereby reduced, protecting the photosynthetic apparatus from photodamage. On the other hand, under reducing conditions, the thiyl radical is converted to a thiol group (or thiolate), and therefore, no excitation quenching takes place.

In contrast to the FMO protein, IsiA has only one cysteine, making it even more crucial in such a quenching process. In this study, we performed site-directed mutagenesis to replace the unique cysteine (C260) in IsiA with a valine. The essentially identical absorption spectra of C260V and WT IsiA (as well as WT PSI-IsiA and PSI-C260V IsiA) demonstrate that C260V IsiA maintains all of the Chl *a*-binding pockets with their Chl *a* molecules, suggesting that C260V IsiA is properly folded. In addition, the Chl *a* Q_y_ absorption bands of both WT and C260V IsiA have a maximum at 670.8 nm, and those of both WT and mutant PSI-IsiA have a maximum at 673.8 nm (Fig. 1 C and D), in good agreement with previous studies (17, 47). While subtle structural changes might have occurred in the C260V IsiA protein, these results suggest that we successfully obtained well-folded free C260V IsiA and PSI-C260V IsiA proteins in the mutant strain.

According to the recent description of the molecular structure of IsiA in *Synechocystis* sp. PCC 6803 (PDB accession number 6NWA) at a 3.5-Å resolution (19), the thiol group of C260 (chain q) lies close to the conserved Chl *a*_5_ (505.q), Chl *a*_6_ (506.q), and Chl *a*_14_ (514.q). In particular, the edge-to-edge distances from Chl *a*_5_ and Chl *a*_6_ are 5.7 Å and 3.7 Å, respectively. These distances are short enough to facilitate electron transfer between these Chl *a* molecules and C260, the basis for the proposed Cys-mediated excitation quenching mechanism (32). The more recent and higher-resolution structures of IsiA from *Synechococcus* sp. PCC 7942 (PDB accession number 6KIF) and Thermosynechococcus vulcanus (PDB accession number 6K33) also exhibit similar associations between these conserved chlorophylls and the unique Cys residue (33, 34).

Cyanobacteria are oxygenic organisms, and unlike FMO, under physiological conditions, IsiA is expected to be in an oxidizing environment. We previously reported that the Chl *a* fluorescence lifetime of IsiA is extended with the addition of a reducing agent (31) owing to the conversion of its thiyl radical to a thiol group under reducing conditions. This prevents the transfer of excited electrons from Chl *a* to the thiyl radical, thus decreasing quenching (32). Our current study shows that the fluorescence decay lifetime of Chl *a* in C260V IsiA is even longer than that of WT IsiA under reducing conditions (Fig. 3), conclusively demonstrating that quenching in IsiA is critically dependent on C260.

In the PSI-IsiA supercomplex, IsiA functions as an accessory antenna that absorbs light energy and transfers excitation to the reaction center of PSI. Our results are consistent with those of previous studies, showing that the energy transfer from IsiA to PSI is rapid and efficient (20, 21, 24). Moreover, when excited at 660 nm, mutant PSI-C260V IsiA and WT PSI-IsiA have identical fluorescence decay traces at 684 nm and 720 nm (Fig. 4), indicating identical excitation energy transfer processes in both samples. These findings showed that mutant C260V IsiA was still capable of transferring excitation energy to PSI and served as an accessory antenna for PSI.

### Physiological consequences of the C260V modification.

Previous studies showed that IsiA is essential for the survival of *Synechocystis* sp. PCC 6803 and *Synechococcus* sp. PCC 7942 under iron-deficient conditions and under high light (12, 26, 41, 48). It has been suggested that these cells cannot survive without IsiA mainly due to the photodamage caused in its absence (12, 26, 41, 48). Our spectroscopic data showed that mutant C260V IsiA no longer quenches excitation energy (Fig. 3) but still functions as an efficient light-harvesting antenna for PSI (Fig. 4). We then determined how this single amino acid substitution affects the physiology of the mutant cells.

### (i) Under iron-replete conditions.

As discussed above, the availability of iron in the cells has a profound effect on the expression of the *isiA* gene. When grown under low light with sufficient iron, the absorption spectra of the C260V mutant and WT cells were almost identical (Fig. 5A), and IsiA was absent from both cultures (Fig. 5B). Under high light, even with sufficient iron, IsiA was induced, as confirmed by spectroscopic (Fig. 5) and immunoblot (Fig. 6C) analyses of the WT and mutant cells. In fact, the increase in the Chl *a* content in the WT under high light was due to the significant expression of IsiA (Fig. 6A and C). In contrast, the C260V mutant showed only a slight increase in the Chl *a* content under the same conditions because of its much lower IsiA content. Given that C260V IsiA cannot quench excitation energy, it is likely that the lower IsiA content in the mutant strain under high light is caused by photodamage.

The growth rates of both strains under low light were nearly identical, but distinct differences in growth patterns were observed. In contrast to the WT, the C260V mutant cells exhibited an initially higher growth rate immediately after inoculation, and no lag phase was observed as is typically seen for the WT (Fig. 7A). Furthermore, under high-light conditions, the C260V mutant grew faster than the WT (Fig. 7B). Appropriate manipulation of photoprotection has been considered to be one of the attractive approaches to improve photosynthetic yield (49). It has been shown that by accelerating recovery from photoprotection or alleviating various photoprotective mechanisms, the growth yields of plants and algae can be substantially improved (50–52). As discussed above, mutant C260V IsiA could serve as an efficient light-harvesting antenna for PSI without any quenching and thus improve cell growth under iron-replete and high-light conditions.

### (ii) Under iron-depleted conditions.

In the absence of iron, both the WT and mutant strains showed a blue shift of the Chl *a* Q_y_ absorption band, indicating the presence of IsiA under both low- and high-light conditions (Fig. 5C). Under high light, there was a significant reduction in the Chl *a* Q_y_ absorption band in the C260V mutant, indicating a lower Chl *a* content in these cells. As discussed previously, a proposed function of IsiA is to maintain the cellular Chl *a* content in iron-deficient environments and help the cells recover once iron becomes available (43–45, 53). Our data show that, compared to the C260V mutant, WT cells are better equipped to maintain their cellular Chl *a* content under high light. Furthermore, under high light, the photoactive PSI and IsiA contents in the C260V mutant strain were significantly lower (Fig. 6B). Because C260V IsiA is unable to quench excitation energy, it is likely that the loss of PSI and IsiA under high light is due to severe photodamage in this mutant strain. In fact, in a strictly iron-free medium, under high light, the C260V mutant exhibited initially fast growth but then bleached out (Fig. 7B). Evidently, the C260V mutant was more light sensitive under iron-depleted conditions, and a fully functional IsiA is necessary for the cells to survive high light in iron-depleted environments.

In summary, we have demonstrated that the C260V mutation abolishes the excitation energy quenching ability of IsiA, confirming the critical role of this unique cysteine residue in the quenching process. Our results further showed that when grown under stringent iron deficiency and high light, the mutant strain is more light sensitive, demonstrating that the cysteine residue in IsiA is crucial for the survival of cyanobacterial cells in such extreme environments. We also determined that C260V IsiA serves as an efficient light-harvesting antenna for PSI. Faster growth was observed in the C260V mutant when grown in the presence of iron under high light. This suggests that the single-amino-acid change may not interfere with other IsiA functions, and in fact, light energy utilization may become more efficient in the mutant cells due to the removal of the relevant energy quenching process. Elucidation of this novel Cys-mediated photoprotective quenching mechanism in an oxygenic photosynthetic organism raises the intriguing possibility of the occurrence of similar mechanisms in plants and algae. Our findings also provide the framework for engineering such an energy dissipation process in other natural as well as artificial Chl antenna proteins to modulate photosynthetic productivities under diverse environmental conditions.

## MATERIALS AND METHODS

### Mutant construction.

To generate the C260V strain, the mutation was introduced with the CRISPR/Cas12a (Cpf1) system reported previously (36). The editing plasmid was constructed by cloning the annealed oligonucleotides and the guide RNA (gRNA) targeting the *isiA* sequence into the AarI site on the pSL2680 vector. The repair template was constructed by Gibson assembly to clone two 900-bp homology regions, including the mutation at the protospacer-adjacent motif (PAM) sequence and the cysteine coding sequence, into the KpnI site on the editing vector. The resulting plasmid, pSL2854, was verified by sequencing and transferred to WT *Synechocystis* cells using the Escherichia coli strain containing the pRL443 and pRL623 plasmids in triparental conjugation (54). The resulting colonies were repatched three times onto BG11 plates containing 10 μg/ml kanamycin. Mutations were verified by sequencing. The verified colonies were grown to stationary phase in BG11 without antibiotics, diluted 1,000 times, and grown to stationary phase again. This process was repeated several times to cure the editing plasmid. BG11 plates with and without kanamycin were used to screen the kanamycin-sensitive colonies, which had lost the editing plasmid. Such a kanamycin-sensitive strain was used as the markerless C260V mutant.

A plasmid to generate the C260V-His strain was constructed by replacing the kanamycin resistance gene in the plasmid that was used to generate an IsiA-His strain (31) with a gentamicin resistance gene and introducing the site-specific mutation into one of the homologous arms. This plasmid was constructed by Gibson assembly (55), using the DNA fragments amplified by PCR. The resulting plasmid, pSL2973, was verified by sequencing. The IsiA-His strain was transformed, and the transformants were selected for growth on gentamicin. Segregation of the C260V-His strain was confirmed by PCR.

### Culture growth conditions and thylakoid membrane preparation.

Wild-type and C260V *Synechocystis* cells were grown photoautotrophically in BG11 under 30 μmol photons m^−2^ s^−1^ of white light at 30°C. After 5 days, cells were harvested and washed three times with YBG11−Fe, a modified medium without any added iron (31, 56). The washed cells were adjusted to the same optical density at 730 nm of 0.05 and grown under 200 μmol photons m^−2^ s^−1^ (low light) or 800 μmol photons m^−2^ s^−1^ (high light). For iron-starved liquid cultures, BG11 was replaced with YBG11−Fe with or without the addition of the chelator deferoxamine (DFB) to a final concentration of 50 μM, depending on the experimental settings. The OD_730_ was continuously recorded every 10 min over the course of the growth experiments. After 3 days of growth, cells were harvested and counted. One milliliter of each culture was used to obtain the absorption spectra, and the rest of the cultures were divided based on the same cell number, resuspended in RB (50 mM morpholineethanesulfonic acid [MES]–NaOH [pH 6.0], 10 mM MgCl_2_, 5 mM CaCl_2_, 25% glycerol), and then stored at −80°C for future use.

The cells were thawed on ice prior to thylakoid membrane extraction. Cells were broken by bead beating as described previously (57, 58), with the following modifications. The thawed cells and 0.17-mm glass beads were loaded into a prechilled Eppendorf tube at a 1:1 ratio of the cell suspension to glass beads. Cells were then broken using 10 break cycles, with each cycle consisting of 1 min of homogenization in a vortex mixer followed by 1 min of cooling. Cell homogenates were centrifuged at 30,000 × *g* for 15 min and washed with RB once. The resulting pellet was resuspended in RB, solubilized with β-d-dodecyl maltoside (DDM) (final concentration of 1%), and incubated on ice in the dark with gentle stirring for 30 min. The sample was then centrifuged at 30,000 × *g* for 30 min, and the resulting supernatant, the solubilized thylakoid membranes, was stored at −80°C for future use.

### Cell counting.

Cell cultures were grown in MC-1000 multicultivators in BG11 and YBG11−Fe under 200 or 800 μmol photons m^−2^ s^−1^ as mentioned above. The cells were harvested after 3 days and diluted to an OD_730_ of 0.01, and the cells were counted on an automated cell counter (Cellometer Vision; Nexcelom) with further manual curation to improve accuracy.

### Photoactive PSI content.

Cells were harvested after 3 days and adjusted to the same cell numbers. With the addition of 10 μM 3-(3,4-dichlorophenyl)-1,1-dimethylurea (DCMU) and 20 μM dibromothymoquinone (DBMIB), which block linear and cyclic electron flow, respectively, the absorbance at 705 nm of P700 in each sample was recorded for 5 s under saturating light on a JTS-10 pump probe spectrophotometer (Bio-Logic Instruments). A molar extinction coefficient of 70 mM^−1^ cm^−1^ for P700 was used to estimate the photoactive PSI content.

### SDS-PAGE and immunoblot analysis.

Solubilized thylakoid membranes of C260V and WT strains were prepared as mentioned above. The solubilized thylakoid membranes were fractionated by denaturing sodium dodecyl sulfate polyacrylamide gel electrophoresis (SDS-PAGE) and analyzed by protein immunoblotting as described previously (59, 60). Fractionated proteins were blotted onto polyvinylidene difluoride (PVDF) membranes. IsiA and D2 were identified by using specific antisera and visualized by using enhanced chemiluminescence reagents (WestPico; Pierce) on an Odyssey Fc imager (Li-Cor Biosciences, USA). The relative protein content was estimated by using Image Studio (Li-Cor Biosciences, USA) based on the chemiluminescence signals of the samples.

### Pigment content estimation.

The Chl *a* concentration was estimated by a methanol extraction method (61). To determine the phycobilin concentration, the absorption spectra of the cultures were obtained on a DW2000 spectrophotometer (Olis, USA), using the equation phycobilin content (milligrams per milliliter) = 0.139 × (*A*_620_ − *A*_730_) − 0.0355 × (*A*_678_ − *A*_730_), where *A*_620_, *A*_678_, and *A*_730_ are the absorbances at 620, 678, and 730 nm, respectively, in a 1-cm path length (62–64).

### Protein complex purification.

A C260V-His liquid culture was grown in YBG11−Fe with the addition of 5 mM deferoxamine under 30 μmol photons m^−2^ s^−1^ at 30°C for 2 weeks to induce *isiA* expression as described previously (31). The solubilized thylakoid membranes were prepared as described above and then used to purify PSI-IsiA and IsiA protein complexes using nickel affinity chromatography and rate-zonal centrifugation (31). After 18 h of ultracentrifugation, fractions were collected. The first and fourth green bands from the top of the gradient contained IsiA and PSI-IsiA supercomplexes, respectively (Fig. 2), as determined by immunoblot and spectroscopic analyses.

### Steady-state and time-resolved spectroscopy.

Steady-state absorption spectra were recorded using a UV-1800 spectrophotometer (Shimadzu). Time-resolved fluorescence (TRF) experiments were carried out using two different setups. For the recording of images of fluorescence profiles of PSI-IsiA supercomplexes at 77 K, a setup based on a Hamamatsu (Japan) universal streak camera described previously (65) was used. Single-wavelength traces for the fluorescence decay of IsiA samples at room temperature were recorded using a standalone Simple-Tau 130 time-correlated single-photon counting (TCSPC) system from Becker & Hickl (Germany). Both setups were coupled to an ultrafast laser system (Spectra-Physics, USA) as described previously (66). The frequency of the excitation pulses was set to 8 MHz, corresponding to ∼120 ns between subsequent pulses. To minimize the detection of scattered light from the excitation beam, a long-pass 665-nm filter was placed at the entrance slit of the spectrograph/monochromator. The integrity of the samples was examined by monitoring the real-time photon count rate over the time course of the experiment. The samples were resuspended to an absorbance of ≤0.1 at the Chl *a* Q_y_ band, and the emission signal was recorded at a right angle with respect to the excitation beam. The excitation beam set to 640 nm, with a photon intensity of ∼10^10^ photons/cm^2^ per pulse, was depolarized and focused on the sample in a circular spot with a diameter of ∼1 mm. Fluorescence emission was recorded at 684 nm.
